# A Laboratory Guide

**Published:** 1890-04

**Authors:** 


A LABORATORY Guide in Urinalysis and Toxicology. By R. A. Witthaus, A. M., M. D:, Piofessor of Chemistry and Physics in the medical department of the city of New York, etc. Second edition. Wm. Wood & Co., New York.

This is a nice little book for students and practitioners. It is illustrated and has alternate blank pages for notes. B.

